# Interprofessional Consensus Regarding Design Requirements for Liquid-Based Perinatal Life Support (PLS) Technology

**DOI:** 10.3389/fped.2021.793531

**Published:** 2022-01-19

**Authors:** M. Beatrijs van der Hout-van der Jagt, E. J. T. Verweij, Peter Andriessen, Willem P. de Boode, Arend F. Bos, Frank L. M. Delbressine, Alex J. Eggink, Jan Jaap H. M. Erwich, Loe M. G. Feijs, Floris Groenendaal, Boris W. W. Kramer, A. Titia Lely, Rachel F. A. M. Loop, Franziska Neukamp, Wes Onland, Martijn A. Oudijk, Arjan B. te Pas, Irwin K. M. Reiss, Mark Schoberer, Ralph R. Scholten, Marc E. A. Spaanderman, Myrthe van der Ven, Marijn J. Vermeulen, Frans N. van de Vosse, S. Guid Oei

**Affiliations:** ^1^Department of Obstetrics and Gynecology, Máxima Medical Centre, Veldhoven, Netherlands; ^2^Department of Biomedical Engineering, Eindhoven University of Technology, Eindhoven, Netherlands; ^3^Department of Electrical Engineering, Eindhoven University of Technology, Eindhoven, Netherlands; ^4^Department of Obstetrics and Gynecology, Division of Fetal Therapy, Leiden University Medical Center (LUMC), Leiden, Netherlands; ^5^Department of Neonatology, Máxima Medical Centre, Veldhoven, Netherlands; ^6^Department of Applied Physics, School of Medical Physics and Engineering Eindhoven, Eindhoven University of Technology, Eindhoven, Netherlands; ^7^Division of Neonatology, Department of Perinatology, Radboud University Medical Center, Radboud Institute for Health Sciences, Amalia Children's Hospital, Nijmegen, Netherlands; ^8^Department of Neonatology, University Medical Center Groningen, University of Groningen, Beatrix Children's Hospital, Groningen, Netherlands; ^9^Department of Industrial Design Engineering, Eindhoven University of Technology, Eindhoven, Netherlands; ^10^Department of Obstetrics and Gynecology, Erasmus Medical Centre, Rotterdam, Netherlands; ^11^Department of Obstetrics and Gynecology, University Medical Center Groningen, University of Groningen, Groningen, Netherlands; ^12^Department of Neonatology, Utrecht University Medical Center, Utrecht, Netherlands; ^13^Department of Neonatology, Maastricht University Medical Center (MUMC), Maastricht, Netherlands; ^14^Department of Obstetrics and Gynecology, Utrecht University Medical Center, Utrecht, Netherlands; ^15^Institute for Applied Medical Engineering and Clinic for Neonatology, University Hospital Aachen, Aachen, Germany; ^16^Department of Neonatology, Amsterdam UMC, Amsterdam, Netherlands; ^17^Amsterdam Reproduction and Development Research Institute, Department of Obstetrics, Amsterdam UMC, University of Amsterdam, Amsterdam, Netherlands; ^18^Department of Neonatology, Leiden University Medical Center (LUMC), Leiden, Netherlands; ^19^Department of Neonatology, Erasmus Medical Centre, Rotterdam, Netherlands; ^20^Department of Obstetrics and Gynecology, Radboud Medical Centre, Nijmegen, Netherlands; ^21^Department of Obstetrics and Gynecology, Maastricht University Medical Center (MUMC), Maastricht, Netherlands; ^22^Care4Neo Foundation, Rotterdam, Netherlands

**Keywords:** perinatal life support, artificial placenta, AAPT, user perspectives, design implications, value-sensitive design

## Abstract

Liquid-based perinatal life support (PLS) technology will probably be applied in a first-in-human study within the next decade. Research and development of PLS technology should not only address technical issues, but also consider socio-ethical and legal aspects, its application area, and the corresponding design implications. This paper represents the consensus opinion of a group of healthcare professionals, designers, ethicists, researchers and patient representatives, who have expertise in tertiary obstetric and neonatal care, bio-ethics, experimental perinatal animal models for physiologic research, biomedical modeling, monitoring, and design. The aim of this paper is to provide a framework for research and development of PLS technology. These requirements are considering the possible respective user perspectives, with the aim to co-create a PLS system that facilitates physiological growth and development for extremely preterm born infants.

## Introduction

Preterm birth is the leading cause of perinatal and neonatal mortality and life-long morbidity world-wide (1). After birth, especially extremely preterm born infants (<28 weeks of gestation) face a challenging transition from fetal to neonatal physiology due to organ immaturity (2). The transition is often complicated by a variety of (sudden) incidents, including heat loss from an immature skin, the necessity of respiratory support, disrupted circulatory adaptations, nutrition deficiency and infections due to an immature immune system and invasive procedures (3, 4). Current treatment requires preterm initiation of body functions for which the respective organs are not yet prepared. This affects primarily the lungs, responsible for gas-exchange (often dependent on mechanical ventilation); the heart, responsible for tissue perfusion and oxygenation; the gut, needed for energy and nutrition; and the brain, with high vulnerability for cerebral hemorrhage (3–5). Consequently, despite the clinical advances at neonatal intensive care units (NICU), still too many extremely preterm but viable infants will suffer permanent health complications.

For decades, researchers have been looking for liquid-based incubators to mimic intra-uterine life for premature new-born infants to prolong fetal physiology (6, 7). Also, the concept of ectogenesis has been described by numerous authors in the beginning of the 20th century (8), and a first patent was filed in 1955 (9).

Based on current scientific advances, it is likely that, following thorough clinical research, PLS technology will ultimately be introduced into clinical practice (10), as an alternative treatment option to conventional neonatal care (11). Research groups in both the US, and in Australia and Japan, recently presented promising pre-clinical results for (extreme) preterm born lambs (12–14). As PLS technology is still developing, a vision on both co-creation of PLS technology and on clinical application of PLS is required, as this will determine design requirements, and also societal acceptance. Therefore, a value-sensitive design approach is preferred (15, 16).

On the one hand, PLS technology yields exciting health promises for mothers and fetuses at risk, as fetal and maternal treatment might now be individually optimized, rather than facing trade-offs between optimizing imperfectly aligned maternal and fetal outcomes (17). Indeed, when successful, i.e., when PLS-based treatment would allow human fetal development and organ maturation as *in utero*, (life-long) complications following preterm birth and neonatal (intensive) care could be prevented (12–14). On the other hand, the technology raises important societal-ethical and legal issues (17–21). Recently, we have established a consortium of healthcare professionals, designers, ethicists, researchers and patient representatives to contribute to the development of PLS technology. In this paper, we present the consensus opinion of this consortium, and propose our vision on the design requirements of PLS-based development. This will provide a framework in which we think this research should be carried-out, based on our vision that PLS-based treatment should be an evolutionary extension of future treatment options in neonatal critical care, for a patient group that presently is already considered eligible for neonatal treatment. Moreover, prior to any form of clinical testing with humans, all potential technical hurdles have to be addressed within this framework. It is of the utmost importance that any development in this field is actively accompanied by socio-ethical debate, rather than that a fully developed technology is being introduced without ongoing debate. To this end, a roadmap on the ethical development of PLS technology has been recently presented by Verweij et al. (16).

## Key Elements

### PLS

PLS refers to application of perinatal life support systems for “developing fetuses” outside the womb, prior to the physiological transition from fetal to neonatal physiology as induced by birth (17, 22). This transition is marked by a cascade of physiological events that among other result in the initiation of breathing and the re-routing of blood flows to accommodate lung circulation as permanent replacement of umbilical and placental circulation (23). To utilize PLS, the initiation of this physiological cascade has to be prevented when the infant leaves the intra-uterine environment, in order to allow further growth and maturation using an artificial placenta (12–14). PLS thus takes over maternal and placental physiological functions, i.e., a form of artificial amnion and placenta technology, also referred to as AAPT (22). By definition, application of PLS implies that the infant has fluid-filled lungs, and/or that the infant is immersed in liquid. It also implies that oxygenation is secured through external oxygen delivery to the infant's blood, thereby bypassing its lungs.

### PLS Transfer Procedure

The transfer of the fetus from the womb to the PLS system has to take place without inducing the physiological cascade of fetal to neonatal transition. It therefore requires fast vascular access through the umbilical vessels to enable connection of the infant's circulation to the artificial placenta (12, 13, 24). Reported animal studies therefore performed a cesarean section, as labor and spontaneous birth set off the cascade of processes in preparation of extra-uterine life (12, 13). For a successful transfer to the PLS system, the transition to neonatal physiology has to be prevented through a safe childbirth procedure for both mother and fetus. Hence, PLS-based treatment after vaginal birth will remain a possibility if fetal breathing and umbilical vascular spasm can be prevented, contamination can be contained and/or fetal infection can be treated (24).

### The Perinate

The infant subject to PLS clearly cannot be categorized based on either the characteristics of a fetus or a neonate: it has been “born-by location change,” but not yet “born-by-physiology-change” (22). The term “gestateling” has been introduced as a definition for an individual with fetal physiology being gestated outside the human body (17), and this has been adopted by others (22). However, this term also refers to (as yet completely hypothetical) cases of both complete ectogenesis and ectogenesis before the threshold of viability. Therefore, we introduce *perinate* as a subcategory of the gestateling, as this term reflects the perinatal stage the infant is in: “peri” (around) “natus” (birth, born), and thus to mark the temporary and specific purpose of perinatal incubation after (extreme) preterm birth as an alternative to perinatal and neonatal care. During a second transition, the perinate is weaned from the perinatal life support and thus becomes a neonate. This involves a complete transition from fetal to neonatal physiology, comparable to normal birth.

## Vision

### PLS Technology Requirements

PLS-based treatment should not be considered as replacement therapy for pregnancy (22), but as an alternative to state-of-the-art neonatal care (17). We envision a PLS environment inspired by, and mimicking the natural womb as much as possible to provide an environment for the perinate that meets all physiological requirements related to the not yet “born-by-physiological-change” principle (22). This implies that design requirements will as much as possible align with the natural environment. Therefore, we propose a perinate-centric perspective that also values the criteria from the other user groups: mother, family and healthcare professionals.

### Perinate's Perspective

From the perspective of the perinate, PLS should be unobtrusive and non- or minimal invasive, and should provide a similar sensation as intra-uterine life. Ideally, the perinate should not be able to differentiate between the intra-uterine and the PLS environment, such that optimal physical, psychosocial and mental health development is enabled and stimulated. To meet this criterium, a set of goals can be defined:

- Fetal physiology needs to be preserved. Hence, the umbilical cord should be the single access point to the fetus, and the placental function has to be mimicked.
- The perinate should receive equal sensory input as during intra-uterine life. This includes stimuli originating from both inside and outside the maternal body, by providing interaction with the surrounding: e.g., auditory and motion stimuli to “experience” the presence of the mother and other family members, either (in)direct or through simulation.
- The perinate should show normal growth and development, expressed not only as physical growth, but also as normal biomarker values (e.g., blood composition), autonomous nervous system development and fetal behavior to facilitate normal psycho-social development.

These goals align with current views presented in literature, displaying that the physiologic needs of the subject of PLS-based treatment are equal to the physiologic needs of an unborn fetus (17–22, 25–27).

### Maternal Perspective

PLS will have a complex and major impact on the mother, that goes beyond the experience of giving extreme preterm birth. She might experience a discrepancy as she is not pregnant anymore, while her child is not yet born but being incubated using PLS. As PLS by definition takes over her physiologic function during gestation, special attention is needed for her physical and psychological needs during both the transfer to the PLS system and the perinate's stay in the PLS system, to minimize maternal stress and facilitate bonding (28). Also, a woman cannot be forced to undergo a cesarean section, even if this will save the life of the unborn child (19, 29, 30). This yields the following criteria:

- The use of PLS should not be restricted to clinical procedures based on cesarean section, but also be compatible with vaginal birth.
- The mother should be enabled to transfer normal, pregnancy-related stimuli to the perinate, such as maternal movements, sounds, endocrine stimuli, and vocal stimuli.
- The mother should be fully supported in her physiological needs and needs to be supported with the after-effects of delivery, such as the onset of lactation and recovery.
- Maternal psychological aspects need to be addressed, including a positive attitude that despite a medical necessity for using PLS, the infant can develop as much as possible in a physiological manner. In addition, attention has to be paid to avoid her having a feeling of guilt toward her baby for not continuously being present during this important developmental stage. We envision to differentiate carefully between functions of the placenta and role of the mother. The latter is much more complex and needs to be supported in all aspects.

Although PLS-based therapy is not considered as a replacement for pregnancy, it can be considered to provide the mother with an option to experience the presence of the perinate, albeit simulated, by using technology e.g., to sense movements of the perinate.

### Family Perspective - Family-Centered Care

The other parent and siblings of the perinate are also affected by the use of PLS, especially with respect to the important process of bonding and the psychological impact. Hence, PLS technology should facilitate and stimulate bonding by allowing other family members to interact with the perinate, whereas psychological aspects need to be addressed during the care process. Hence, PLS-based treatment should allow and encourage active parental involvement, even though direct interaction might be challenging regarding its potential interference with normal growth and development and risk for infection. When considering (discontinuation of) PLS treatment, this should be accompanied with timely and respectful counseling and shared-decision-making to prevent moral pressure and therapeutic misconception (31).

### Healthcare Professional Perspective

PLS technology should facilitate healthcare professionals in caring for the perinate. Hence, the following sub-goals can be defined:

- PLS technology should provide healthcare professionals with information: clear, correct and timely data, where signals are converted into meaningful and interpretable information, using a clinical decision support system based on digital twins of both the perinate and the PLS system, and provide early warning (32).
- PLS technology should provide suitable access points for clinical care, in a least complex and safe manner, to prevent medical and patient safety errors.
- Healthcare professionals need to be able to interfere and override PLS technology in case of emergency. The final responsibility rests with the clinician in charge, not the system.
- The care team should include people with knowledge on clinical care, physiology, technical care, growth and development, perinatology and neonatology. The team should have one medical, and one technical responsible leader, with joint final responsibility.

## PLS Technology As New Approach to Perinatal Care

### Design Implications

The different user perspectives all underline that PLS technology should mimic and emphasize the natural functions of the physiological womb. The following design implications have been derived by stakeholder input, i.e., of healthcare professionals, designers, ethicists, researchers and patient representatives, based on this integral vision. The design implications should drive technological feasibility, not the other way around. Indeed, innovations follow clinical and technical challenges.

#### Placenta

Due to its vascular architecture, the placenta cannot be detached from the uterus for use in an extrauterine environment but has to be replaced by an artificial organ. The most vital placental function is gas exchange. Therefore, oxygenators, connected to the circulation via the umbilical vessels in experimental research, have been used and referred to as artificial placentas since the late 1950s (6, 7). Technological advance has led to a reduction of size and resistance, thereby allowing to use pumpless extracorporeal circuits (12, 14, 33, 34). Other important placental functions include metabolic waste products removal, hormones formation, supply of micro- and macronutrients, and the transfer and accumulation of (maternal) antibodies into the fetal blood (35). Of these, the elimination of urinary excreted substances would be most likely technical feasible with current medical technology (36). Technical challenges lie within the objective to minimize activation of coagulation and inflammation induced by foreign surfaces (37), and in appropriate sizing.

#### External Stimuli

*In utero*, sensory stimuli are essential for physiologic development of the senses (38). PLS technology should offer natural sensory input to the perinate as to stimulate normal growth and (neurological) development. Sensory stimuli, both originating from the maternal biological environment as from the outside world, should be applied to the perinate using targeted technology. Individualized inputs can be either simulated, pre-recorded or real-time, and can include maternal physiological sounds (like heartbeats), maternal movements, uterine contractions, diurnal rhythm, maternal endocrine factors, etcetera.

#### Chamber-Within-Chamber Design

We envision a closed system that stretches with the perinate's growth, such that the perinate has little perception of the differences between the real and mimicked womb. As every fetus has its unique intra-uterine environment, a chamber-within-chamber design for the PLS system should be provided. The inner chamber provides a liquid-based environment with sterile artificial amniotic fluid, while the outer chamber can be used to apply auditory, visual and tactile stimuli in a controlled environment specific for each perinate.

#### Medical Treatment

PLS technology should allow medical treatment and administration of drugs, appropriate for the perinate's needs. Administration and access routes can be multiple: artificial placenta, amniotic fluid, etcetera. For manual access to the perinate, e.g., for emergency delivery or fetal therapeutic treatment such as surgery, the inner chamber should be accessible, and a procedure to prevent any infection provoked by opening the chamber should be in place.

#### Monitoring

Continuous and tailored monitoring of the perinate's growth and well-being contributes both to fine-tuning of the system's life support functions and in decision-making. Monitoring includes electrocardiography, electroencephalography, movements, nutritional status, oxygenation, carbon dioxide exchange, fluid balance, temperature, biomarker and metabolite concentrations, blood count, circulatory and kidney function, using sensor technology and metabolomics analysis. The collected data will provide insights into perinatal physiological and pathophysiological processes, circadian rhythms and the influence of environmental factors like nutrition, light and physical stimuli on perinatal wellbeing.

#### Clinical Transfer Procedures

First transfer: Sterile and smooth birth by cesarean section or vaginal delivery, whereby the perinate is protected against breathing while being transferred from the uterus into an airtight bag. This procedure should be as least interfering with the perinate's perception as possible, as the perinate should experience the PLS system as if no birth has taken place. Also, this procedure should promote best maternal physical and psychological health. Second transfer: Simulated birth when the perinate leaves the PLS system: this transfer finalizes birth and should represent the beneficial effects of an actual birth as much as possible. This could include applying uterine contractions to the PLS system and providing a passage of the infant through a simulated birth canal.

#### PLS Technology Research and Development

Research and development of PLS technology should be driven by the design requirements and is obviously influenced by current technological advances and limitations. Indeed, PLS-based research faces many research challenges, e.g., regarding the abandonment of systemic anticoagulation, the need for a much deeper knowledge of the molecular functions of the feto-placenta-maternal unit, and the inflammatory effects of technical devices. Yet, technological innovation can only follow from a clear vision and related design requirements. While developing working prototypes, thorough testing in a simulated environment with computer models and high-fidelity physical manikins is highly recommended. After such technical validation, animal experiments in established animal models (12, 14, 39–43) can confirm the feasibility to promote a clinical trial.

### Implications for Clinical Care

Clinical care using PLS technology should be carried out on a specific perinatal intensive care unit, in which obstetric high care and neonatal intensive care are integrated in a family-centric approach. PLS-based treatment should be trusted to a specifically trained care team, consisting of medical and technical professionals that work in respectful dialogue with the parents. This may lead to new clinical specialisms, such as a “perineonatologist” with knowledge of maternal, fetal and perinatal physiology; technical specialists; perfusionists; specialized anesthesiologists; specialized nurses; social workers and psychologists. Obviously, research should include thorough *in vitro* and *in silico* testing, animal experiments, and (pre-)clinical trials. Moreover, in-depth evaluation of both short- and long-term clinical outcome of infants treated with PLS-based procedures has to provide evidence on whether PLS-based treatment is superior to current treatment options, before it is widely implemented (11).

## Discussion

PLS-based treatment is an exciting and promising method to limit or even prevent the consequences of extreme preterm birth. When designing PLS technology, the natural womb should as much as possible set the design requirements; and current advances in technology are encouraging. Application of PLS comes with ethical and societal responsibilities, as has been elaborated in a recently presented roadmap on the ethical development of PLS technology (16). Hence, even if all technical challenges would be met, ethical, legal and societal implications should be considered carefully, as the availability of PLS will highly influence societal values and perceptions regarding e.g., pregnancy, childbirth, women and (unborn) babies, but also the moral and legal status of the perinate. Therefore, we advocate stakeholder involvement and a value-sensitive design approach *throughout* the research and development process (16).

Human research ethics for PLS needs further study to address important aspects like patient selection and informed consent (16). Similarly to selecting appropriate patient groups for clinical trials (11), also once clinically implemented this mandates a head-to-head comparison with standard neonatal care. One of the most important aspects will be the counseling strategy (16). PLS could be an answer to the dilemma of the benefits outweighing the risks of continuing pregnancy vs. the health risks involved for either the mother or the infant (17). Yet, first has to be investigated whether PLS-based treatment is superior to the current standard of treatment (11, 44). Without evident proof on the short- and long-term implications of infants being gestated using PLS, great caution is needed. In addition, we strongly urge teams involved in the development of PLS-based treatment to embrace and build on all valuable things learned in conventional antenatal and neonatal care. These include essential concepts as shared decision-making, counseling at the border of viability, family-centered care and the NIDCAP approach to neonatal development (45). Ultimately, knowledge acquired in PLS-based research and clinical care should help to improve standard care as well.

## Data Availability Statement

The original contributions presented in the study are included in the article/supplementary material, further inquiries can be directed to the corresponding author.

## Author Contributions

MH wrote the main manuscript text based on a discussion session with FD, LF, RL, MO, RS, FV, and SO and separate consultation with IR, EV, and MV. All authors provided further input and reviewed the manuscript.

## Funding

This work was funded in part by the European Union *via* the Horizon 2020: Future Emerging Topics call (FET Open), Grant EU863087, project PLS.

## Conflict of Interest

The work of MH, FD, LF, FN, MS, MV, SO, and FV has been funded by the European Union via the Horizon 2020: Future Emerging Topics call FET Open, grant EU863087, project PLS, https://cordis.europa.eu/project/id/863087. MH, MV, and SO are shareholders in Juno Perinatal Healthcare BV, Netherlands. AP is Chair of Scientific Advisory Board of Concord Neonatal BV, for which he receives no compensation, https://concordneonatal.com. He also consults for Fisher and Paykel Healthcare and receives compensation https://www.fphcare.com/en-gb. The remaining authors declare that the research was conducted in the absence of any commercial or financial relationships that could be construed as a potential conflict of interest.

## Publisher's Note

All claims expressed in this article are solely those of the authors and do not necessarily represent those of their affiliated organizations, or those of the publisher, the editors and the reviewers. Any product that may be evaluated in this article, or claim that may be made by its manufacturer, is not guaranteed or endorsed by the publisher.
